# Molecular characterization and expression variation of the odorant receptor co-receptor in the Formosan subterranean termite

**DOI:** 10.1371/journal.pone.0267841

**Published:** 2022-04-28

**Authors:** Paula Castillo, Claudia Husseneder, Qian Sun

**Affiliations:** Department of Entomology, Louisiana State University Agricultural Center, Baton Rouge, Louisiana, United States of America; Monell Chemical Senses Center, UNITED STATES

## Abstract

Subterranean termites live in underground colonies with a division of labor among castes (i.e., queens and kings, workers, and soldiers). The function of social colonies relies on sophisticated chemical communication. Olfaction, the sense of smell from food, pathogens, and colony members, plays an important role in their social life. Olfactory plasticity in insects can be induced by long- and short-term environmental perturbations, allowing adaptive responses to the chemical environment according to their physiological and behavioral state. However, there is a paucity of information on the molecular basis of olfaction in termites. In this study, we identified an ortholog encoding the odorant receptor co-receptor (Orco) in the Formosan subterranean termite, *Coptotermes formosanus*, and examined its expression variation across developmental stages and in response to social conditions. We found that *C*. *formosanus* Orco showed conserved sequence and structure compared with other insects. Spatial and temporal analyses showed that the *Orco* gene was primarily expressed in the antennae, and it was expressed in eggs and all postembryonic developmental stages. The antennal expression of *Orco* was upregulated in alates (winged reproductives) compared with workers and soldiers. Further, the expression of *Orco* decreased in workers after starvation for seven days, but it was not affected by the absence of soldiers or different group sizes. Our study reveals the molecular characteristics of *Orco* in a termite, and the results suggest a link between olfactory sensitivity and nutritional status. Further studies are warranted to better understand the role of *Orco* in olfactory plasticity and behavioral response.

## Introduction

Chemical communication is essential for the regulation of fundamental behaviors in social insects [1]. In termites (order Blattodea), the division of labor in a colony is maintained through a caste system consisting of reproductives (queens and kings), workers, and soldiers. In subterranean termites (family Rhinotermitidae), colonies are typically founded by a pair of dispersal alates (i.e., winged reproductives), which shed their wings, build an underground nest chamber, and rear the first cohort of brood as the primary queen and king [2]. Newly developed workers then take over the tasks of brood care, tunnel in soil to search for food, and perform hygienic activities [3, 4]. Soldiers defend the colony through aggressive behavior toward competitors and predators [5]. The elaborate caste system results from developmental plasticity, which is mainly mediated by the social environment. After hatching from the eggs, the larvae follow either an apterous pathway to differentiate into workers and soldiers, or an imaginal pathway to develop into nymphs and eventually alates [6]. Except for the alates, subterranean termites have poor visual ability and heavily rely on chemicals to organize social activities and detect environmental changes [7]. Pheromones also play key roles in mediating caste differentiation in termite colonies [8–10].

In insects, perception of olfactory cues is primarily performed by the antennae, which carry numerous and diverse sensilla [11]. Olfactory sensilla are characterized by a multiporous surface that allows volatile chemicals from the environment to enter the sensillar lymph. Dendrites of olfactory sensory neurons (OSNs) extend to these hair-like sensilla, and odorant receptors (ORs) are expressed in the dendritic membranes [12]. Through activities of OSNs, odorant signals are transduced into electrical impulses, which are integrated in the antennal lobes and may further project to higher brain centers, such as mushroom bodies, to trigger a behavioral response [12, 13]. The ORs have undergone rapid evolution in insects, facilitating their adaptation to a wide range of ecological niches [12, 14]. While each species possesses a distinct OR repertoire, most species express only one odorant receptor co-receptor (Orco), which is highly conserved across insects [12]. Subunits of Orco form a tetramer arranged around a central pore and are bound together by a small cytoplasmic anchor domain [15]. While ORs confer odorant specificity, they form Orco-OR heterotetramers that compose the odorant-gated ion channels. Therefore, Orco is required for localization of ORs to dendritic membranes and their proper function [15]. Most OSNs express only one unique member of its OR family along with the ubiquitous chaperone Orco [16–18].

The function of Orco has been investigated in many solitary and a few social insects, revealing its role in the regulation of essential behavior and neurodevelopment. For example, mutation of *Orco* gene through CRISPR-Cas9 disrupted larval feeding and adult mating behavior in the domestic silk moth (*Bombyx mori*) [19] and foraging behavior in the hawkmoth (*Manduca sexta*) [20]. Silencing of Orco through RNA interference (RNAi) impaired mating behavior in the olive fruit fly (*Bactrocera oleae*) [21] and responses to aggregation pheromone and food seeking behavior in the white-spotted flower chafer beetle (*Protaetia brevitarsis*) [22]. In addition, expression of *Orco* is required to avoid degeneration of OSNs in the maxillary palps in the vinegar fly (*Drosophila melanogaster*) [23]. *Orco* is also essential for maintaining social behavior and the development of antennal lobes in the clonal raider ant (*Ooceraea biroi*) [24], the Jerdon’s jumping ant (*Harpegnathos saltator*) [25], and the honey bee (*Apis mellifera*) [26]. In Blattodea, *Orco* has been functionally characterized in the German cockroach (*Blatella germanica*) [27], and in the worker caste of two termite species, a fungus-growing termite (*Odontotermes formosanus*) [28] and a subterranean termite (*Reticulitermes chinensis*) [29]. In *B*. *germanica*, the silencing of *Orco* increased their response time to the sex pheromone blatellaquinone and impaired their food-seeking behavior [27]. In termites, the knockdown of *Orco* altered nestmate discrimination in *O*. *formosanus* [28] and affected trail-following and locomotion behavior in both *O*. *formosanus* and *R*. *chinensis* [29]. As numerous studies examine the function of Orco, the expression of *Orco* may be one of the indicators of the olfactory sensitivity and activity in olfaction-related neurogenesis. However, its expression variation during caste development is not examined in social insects.

The olfactory system displays sex dimorphism in many insects and caste polyphenism in some social species, reflecting their differential olfactory capacity and behavioral repertoire [30, 31]. In social Hymenoptera (ants, bees, and wasps), most social behavior is displayed by females (workers and queens), and males are produced for mating purpose only. Correspondingly, these sexes/castes exhibit variation in terms of peripheral sensory structures and chemosensory gene profiles [30, 31]. For instance, the hydrocarbon-sensitive basiconic sensilla, as well as the 9-exon subfamily of ORs, which detects cuticular hydrocarbons for nestmate recognition, are only found in female ants of *O*. *biroi* [31]. Different from social Hymenoptera, hemimetabolous termite castes are comprised of both females and males [6]. Our previous study in the Formosan subterranean termite, *Coptotermes formosanus*, showed that the composition of antennal sensilla varied between reproductive and non-reproductive castes, but not between female and male alates [32]. In the Japanese subterranean termite, *R*. *speratus*, caste-specific chemosensory gene expression profiles are reported [33]. It is unknown, however, if the expression of *Orco* varies during the plastic development of caste system in termites.

Olfactory plasticity is widely observed in animals, which enables them to modify behavioral responses according to physiological state such as age, feeding, and mating status [34]. Long-term environmental conditions and the immediate sensory environment can influence olfactory plasticity [35]. Olfaction can be tuned through changes in chemosensory gene expression, neuromodulators, and endocrinological mechanisms [34]. For example, starvation increases olfactory sensitivity and enhances food seeking behavior in insects, worms, rodents, and humans [36–38]. Changes in chemosensory gene expression were induced by starvation in the oriental fruit fly, *Bactrocera dorsalis* [39], and African cotton leafworm, *Spodoptera littoralis* [40]. In *B*. *dorsalis*, expression of *Orco* was upregulated by a sexual attractant, methyl eugenol [41]. In termites, social environment is important for both caste development and immediate behavioral response [6, 7], but the expression variation of *Orco* in response to environmental conditions has not been investigated. The function of social colonies is maintained by collective activities through pheromonal regulation and nestmate recognition. A number of pheromones with behavioral and/or physiological activities have been identified in termites. For example, soldiers produce a pheromone that modulates worker behavior and worker-soldier differentiation [10], workers release a trail pheromone to recruit nestmates for foraging [42], and the queen produces a pheromone that suppresses nestmate fertility and/or promotes their tending behavior [8, 9]. In addition, cuticular hydrocarbons are important for nestmate and caste recognition [7]. So far, the conserved *Orco* has been identified in five termite species: two subterranean termites, *R*. *speratus* [33] and *R*. *chinensis* [29], a dampwood termite *Zootermopsis nevadensis* [43], a drywood termite *Cryptotermes secundus* [44], and a fungus-growing termite *O*. *formosanus* [28, 29]. However, the role Orco plays in olfactory plasticity remains unclear.

The overall goal of this study was to characterize the *Orco* ortholog and examine its expression variation in *C*. *formosanus*, which is a cosmopolitan invasive species. Since antennae are the primary olfactory organs, and the division of labor in social insects is often associated with olfactory capabilities, we hypothesized that *Orco* expression is tissue-specific and varies across termite castes. In addition, as social behaviors in termites are largely mediated through chemical cues, we further hypothesized that the expression of *Orco* changes in response to social environment. To test the hypotheses, we (i) cloned and sequenced the full-length cDNA of *Orco* in *C*. *formosanus*, (ii) analyzed Orco protein structure in *C*. *formosanus* and its phylogenetic relationship with other insects, (iii) examined the spatial (tissue) and temporal (developmental stage) distribution of *C*. *formosanus Orco*, and (iv) investigated the expression profiles of *C*. *formosanus Orco* under three sets of varying social conditions, including starvation, soldier percentage, and group size.

## Materials and methods

### Insects

Three colonies of *C*. *formosanus* workers and soldiers were collected at Brechtel park in New Orleans, Louisiana (29°54′32″N, 90°00′32″W). These colonies were kept at 25 ± 1°C in clear acrylic containers (38.48 × 45.72 × 22.86 cm), provided with an approximately 4.0 cm layer of organic soil at the bottom and moistened pine wood logs as the food source. The relative humidity (RH) in each container was monitored weekly, and water was added to maintain 80–99% RH. Workers, soldiers, pre-soldiers, and nymphs were obtained from these colonies. Female and male alates were collected using ultraviolet light traps (BioQuip, USA) during the swarm season (May and June) from three populations in Baton Rouge, Louisiana (population A: 30°22′14″N, 91°06′39″W; population B: 30°24’33.3’’N, 91°06’18.5’’W; population C: 30°22’12.2’’N, 91°06’16.8’’W). For RNA sample preparation, the alates were utilized within 16 h upon collection from the field. Heterosexual pairs of alates were allowed to form incipient colonies in Petri dishes (5.0 cm in diameter, Thermo Fisher Scientific, Waltham, MA USA) provisioned with moist filter paper and pine wood chips. These incipient colonies were kept at 27 ± 1°C and 80–99% RH in complete darkness for two months, and eggs and larvae were collected from three colonies.

### Cloning and sequencing of *Orco*

Total RNA from pools of 200 antennae from 100 workers, female, or male alates were extracted using TRIzol reagent (Invitrogen, Waltham, MA, USA) following the manufacturer’s protocol. Then, all samples were treated with DNAse I to remove traces of genomic DNA, utilizing the TURBO DNase kit (Invitrogen), following the manufacturer’s protocol. The quantification of the purified total RNA was performed on a NanoDrop^™^ One spectrophotometer (Thermo Fisher Scientific). To obtain the full-length transcript, RACE-ready cDNA was synthesized with the GeneRacer Kit (Invitrogen) following the manufacturer’s protocol. Gene specific primers (GSPs) for 5’ and 3’ cDNA ends were designed upon conserved regions of *Orco* gene across different termite species (S1 Table), utilizing the NCBI primer designer online tool PrimerBLAST (https://www.ncbi.nlm.nih.gov/tools/primer-blast/). The touchdown PCR reactions were carried out with the Platinum^®^ *Taq* DNA Polymerase High Fidelity (Invitrogen), using the following cycling conditions: initial denaturation at 94 °C for 2 min, followed by 15 cycles at 94 °C for 15 s (denaturation), annealing temperature touchdown from 78 °C to 68 °C for 45 s, and 72 °C for 1 min (extension); then, 20 cycles at 94 °C for 15 s, 66 °C for 45 s, and 68 °C for 1 min. A final extension step was added at 68 °C for 2 min. The PCR products were purified from an agarose gel, using the Zymoclean Gel DNA Recovery kit (Zymo Research, Irvine, CA, USA), and cloned into the pCR^®^4-TOPO^®^ vector (Invitrogen), followed by transformation in the One Shot^®^ TOP10 chemically competent *E*. *coli* cells, according to the manufacturer’s protocol. Transformants were obtained after selection with ampicillin, and the plasmid isolation was achieved by using the QIAprep^®^ Spin Miniprep Kit (Qiagen, Germantown, MD, USA). Then, the sequences in both directions were obtained through Sanger sequencing utilizing the forward and reverse M13 primers at the Genomics Facility of Louisiana State University.

### Structural simulation and phylogenetic analysis

The *Orco* sequence in *C*. *formosanus* was compared with other insect species using BLAST (https://blast.ncbi.nlm.nih.gov/). Identification of the full open reading frame (ORF) and translation into protein was performed with the on-line tool ORFfinder (https://www.ncbi.nlm.nih.gov/orffinder/). Identification of conserved protein domains was carried out using the Simple Modular Architecture Research Tool (SMART) (http://smart.embl.de/) and was confirmed with InterPro scan (https://www.ebi.ac.uk/interpro/). A 2D structure of the putative *C*. *formosanus* Orco protein was predicted with the TMHMM 2.0 online tool (https://services.healthtech.dtu.dk/service.php?TMHMM-2.0), and the graphical representation was generated with TMRPres2D (http://bioinformatics.biol.uoa.gr/TMRPres2D/) following the developer’s recommendations [45]. The 3D structure was predicted using the SWISS-MODEL server (https://swissmodel.expasy.org/) and the Cryo-EM structure of Orco from *Apocrypta bakeri* as a template [15]. Prediction of molecular mass and isoelectric point of the putative Orco protein were estimated with the pI/Mw tool in Expasy (https://www.expasy.org/). Amino acid sequences of Orco from different insect species were downloaded from the NCBI database (S2 Table). Then, protein alignment was performed using the MUSCLE module in the Geneious primer software (Biomatters, Inc, San Diego, CA, USA), which was further utilized for phylogenetic reconstruction through IQ-Tree (http://www.iqtree.org/) using the maximum-likelihood method. Node support values were obtained from 1000 bootstrap replications. The graphic representation of the phylogenetic tree was obtained and adjusted using the iTOL on-line platform (https://itol.embl.de/). The sequence of the pea aphid (*Acyrthosiphon pisum*) was manually selected as the root for the phylogenetic tree.

### Spatial and temporal expression of *Orco*

To determine the spatial expression of *Orco*, its expression levels in antennae, head (without antennae), legs, and carcass (remaining body part) were analyzed in workers, soldiers, and female and male alates collected from three colonies or populations (n = 3 per tissue type). These termites were processed for sample collection within 16 hours upon collection. Termites were anesthetized on ice prior to dissection. To obtain sufficient RNA, 200 antennae were pooled from 100 individuals, and 600 legs were pooled from 100 individuals, along with the heads of 30 individuals and the carcasses of 10. The heads and carcasses of soldiers were not analyzed due to the interference of frontal gland secretion with RNA isolation. For the temporal expression, different developmental stages and castes, i.e., eggs, larvae, workers, pre-soldiers, nymphs, female and male alates, were analyzed. Specifically, pools of 25 to 45 eggs, 7 to 11 larvae (first and second instars), or a whole body of each caste (third instar or older) from three colonies or populations were used (n = 3 per developmental stage/caste).

Tissues or whole bodies were collected directly in TRIzol (Invitrogen). Total RNA isolation and treatment with DNAse was performed as described in section 2.2. The cDNA was synthesized with the SuperScript III first-strand synthesis kit (Invitrogen) from 330 ng of total RNA from each sample, following the manufacturer’s protocol. The relative quantification of *Orco* gene expression was achieved through quantitative real-time PCR (qRT-PCR) using 1 μL of diluted cDNA (4.125 ng/μL) and the PowerUp^™^ SYBR^™^ Green Master Mix (Applied Biosystems) on a QuantStudio 3 real-time PCR system (Thermo Fisher Scientific). A final concentration of 300 nM of the primers listed in S1 Table were utilized for *Orco* and each reference gene. The cycling conditions were: 2 min at 50 °C for UDG activation; 2 min at 95 °C hot start activation; then, 40 cycles of 15 s at 95 °C (denaturation), and 1 min at 60 °C (annealing/extension). Finally, the melting curve analysis to confirm the specificity of the reaction was performed with an initial denaturation step of 15 s at 95 °C, followed by a heating temperature ramp from 60 °C to 95 °C with an increase at 0.05 °C/s. ROX was utilized as the passive reporter.

Four reference genes, the ribosomal protein S18 (*rps18*), ribosomal protein L32 (*rpl32*), elongation factor 1-α (*ef1-α*), and the structural protein *β-Actin* were assessed for their stability across different gene expression analyses in all tested conditions using the BestKeeper Excel-based tool [46]. The selection criteria of the best reference genes by this method consider the following factors: 1) genes with a standard deviation of raw Ct values higher than 1.0 are considered inconsistent, and 2) genes with expression 3-fold over or under their geometric mean Ct should be discarded. Primers for all reference genes were taken from Du, et al. [47] (S3 Table), and primer specificity was determined by the melting curve analysis. Efficiency for each qRT-PCR reaction was determined utilizing the raw amplification data and the LinRegPCR software [48, 49]. The baseline corrected data was used to calculate the relative expression levels of *Orco* using the 2^-ΔΔCt^ method described by Livak and Schmittgen [50]. One stable reference gene, *rps18*, was selected for normalization of the data in these calculations.

### Expression of *Orco* in response to different social environments

Three assays were performed to evaluate the expression of *Orco* in response to starvation, the presence of soldiers, and different group sizes. In all three assays, termites were maintained in Petri dishes (35 mm in diameter) lined with moist filter papers as the food source, and the termites were kept at 27 ± 1°C, 80–99% RH, in complete darkness. For the starvation assay, groups of 50 termites (45 workers and five soldiers) were starved for 0, 5, or 7 days. Three replications were conducted per colony, and three colonies were used (n = 9 per treatment). For the influence of soldiers, groups of 45 workers were maintained with the addition of five soldiers (10%) or without soldiers (0%) for seven days. Six replications (two each from three colonies) were performed. For the test of group size, workers were kept in Petri dishes for seven days as individuals, groups of 15, or groups of 100. Each group size was tested with six replications (two each from three colonies). For each sample, 20 antennae from 10 workers were dissected and collected directly in TRIzol (Invitrogen), and total RNA isolation and gene expression analysis were performed as described in section 2.4. *rps18* was used as the reference gene for qRT PCR analysis (S3 Table).

### Statistical analyses

All statistical analyses were carried out using the R software version 4.1.2 (The R Foundation, Vienna, Austria) [51]. To check the homogeneity of variances and normality of data distributions, Levene’s test and Shapiro–Wilk test were performed, respectively. As data did not meet the assumptions of parametric tests (*P* < 0.05 for Levene’s test or Shapiro–Wilk test), Kruskal–Wallis followed by Dunn’s tests were performed to analyze the expression levels of *Orco* for its spatial and temporal distribution, and in response to different social environments. An alpha level of 0.05 was chosen for all tests performed. All gene expression data were plotted utilizing GraphPad Prism version 9.3.1 (San Diego, California, USA).

## Results

### Bioinformatic analysis of *Orco*

**Fig 1:**
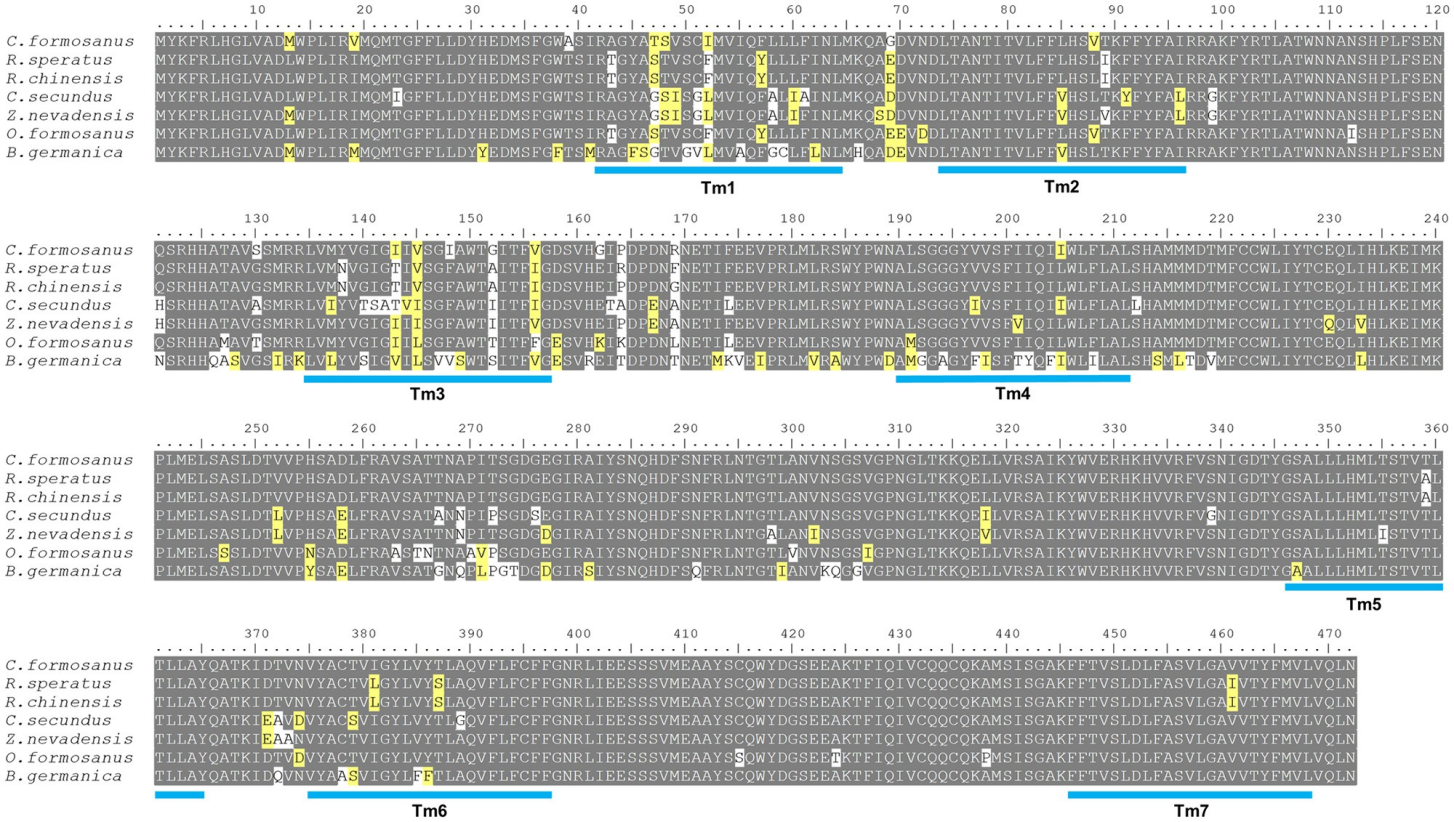
Alignment of Orco proteins in *C*. *formosanus* and other Blattodea species. Conserved regions are shown in gray background, and mismatches above or below 50% are shown in yellow and white backgrounds, respectively. The predicted 7tm_6 transmembrane domains (Tm1-7) are denoted with cyan-colored lines.

Only one isoform of *Orco* was identified in *C*. *formosanus* with an ORF of 1,419 bp in length (GenBank accession number OL845867). It encoded a predicted protein of 472 amino acids (aa) (Fig 1) with a predicted molecular mass of 53.15 kDa and an isoelectric point (pI) of 7.21. The full-length cDNA included a 214 bp 5’ untranslated region (UTR) and an 878 bp 3’ UTR with a poly (A) tail.

Multiple amino acid sequence alignments showed that Orco was highly conserved across termite species (Fig 1). The putative Orco sequence in *C*. *formosanus* shared 94.92% identity with *Orco* in the Chinese subterranean termite *R*. *chinensis* (Rhinotermitidae); 94.70% with the Japanese subterranean termite *R*. *speratus* (Rhinotermitidae); 91.74% with the fungus-growing termite *O*. *formosanus* (Termitidae); 91.95% with the dampwood termite *Z*. *nevadensis* (Termopsidae); 89.19% with the drywood termite *C*. *secundus* (Kalotermitidae); and 82.42% with the German cockroach *B*. *germanica* (Ectobiidae), which is an omnivorous and group-living species.

**Fig 2:**
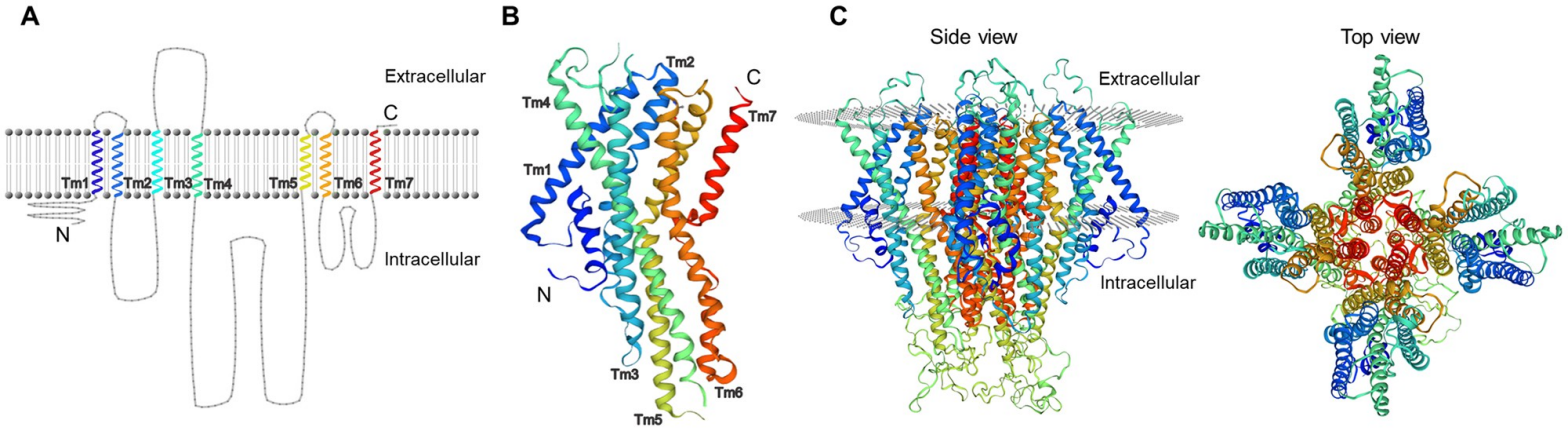
Structure of *C*. *formosanus* Orco protein. (A) 2D model showing 7tm_6 transmembrane domains (Tm1-7), intracellular, and extracellular regions of Orco. (B) 3D structure of an Orco subunit showing all transmembrane regions (Tm1-7). (C) 3D model of Orco’s homotetramer viewed from the side and top (extracellular).

The structural analysis of the putative Orco protein utilizing the TMHMM online tool revealed a short intracellular domain (amino acids 1 to 41) at the N-terminus (probability < 0.2), followed by 7 predicted transmembrane regions that belong to the 7tm_6 domain (Tm1-7) family (probability > 0.85); which are characteristics of odorant receptors (Fig 1). A schematic representation of the 2D structure of Orco with predicted transmembrane, cytoplasmic, and extracellular regions is shown in Fig 2A. The *in silico* simulation for *C*. *formosanus* Orco with SWISS-MODEL rendered a 3D model that shared 63.93% sequence identity with *A*. *bakery* Orco protein. In this model, the transmembrane regions folded as alpha-helixes are shown in Fig 2B for a single Orco subunit. The subunits of Orco were predicted to form a homotetramer, with each transmembrane region assisting in the formation of a channel pore in the center (Fig 2C).

**Fig 3:**
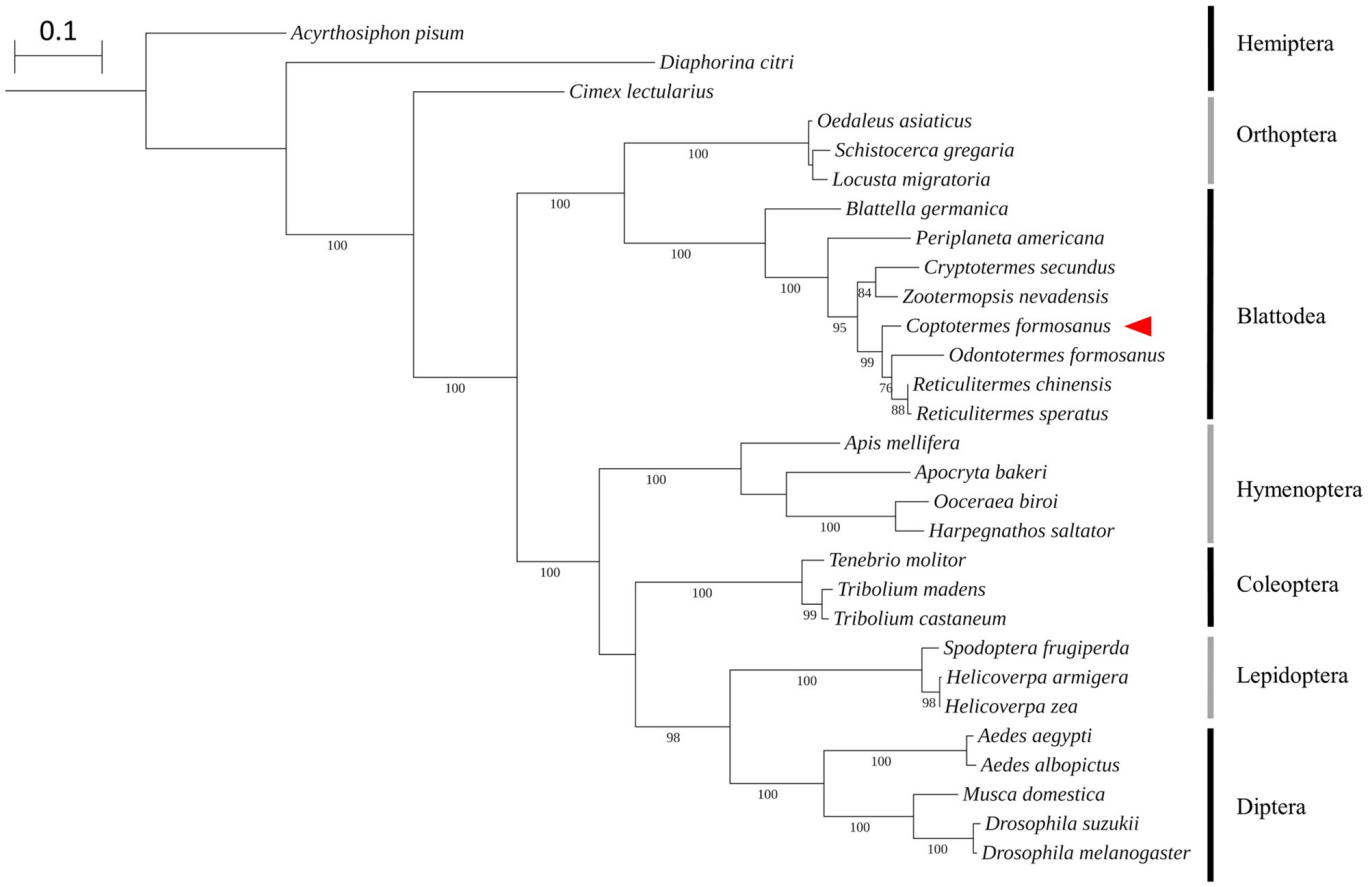
Phylogenetic relationships of Orco across different insect taxa. The maximum likelihood tree was constructed based on the amino acid sequences of Orco in different insect species. Species of focus in this study is indicated with a red arrow. Numbers in each branch correspond to the bootstrap values. The scale bar represents number of substitutions per site.

The phylogenetic analysis showed that all known Orco sequences in termites formed a monophyletic clade, which is closely related to *Blattella germanica* Orco (Fig 3). Based on the phylogenetic reconstruction, the Orco proteins of Blattodea are evolutionary closer to those in Orthoptera than in Hemiptera, Hymenoptera, Coleoptera, Lepidoptera, and Diptera (Fig 3).

### Spatial and temporal expression of *Orco*

**Fig 4:**
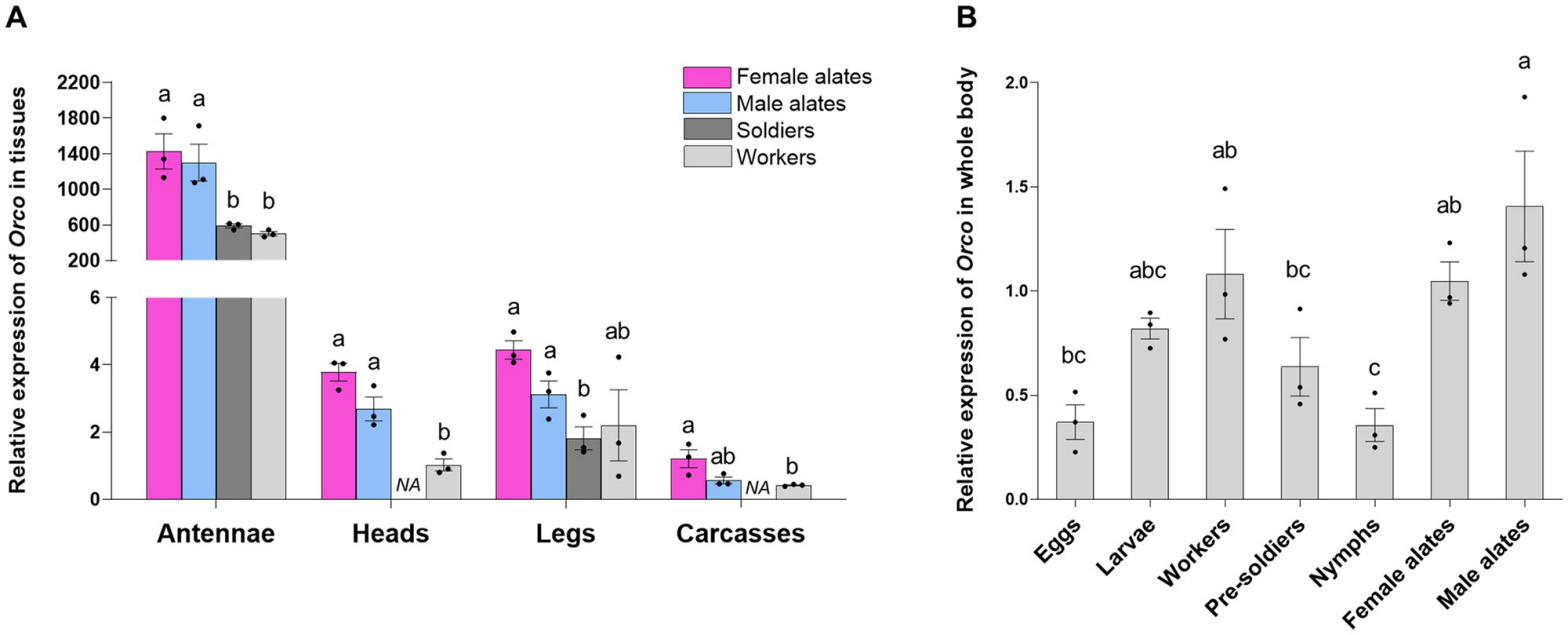
Spatial and temporal expression of *Orco*. (A) *Orco* expression profiles across four tissues in workers, soldiers, female and male alates. Data were comparatively analyzed among castes for the same tissue type. (B) *Orco* expression profiles using the whole body of termites across seven developmental stages. Different letters denote significant differences by Kruskal-Wallis followed by Dunn’s test (*P* < 0.05, n = 3); *NA*: not available. In both graphs, bars represent means ± SE with individual data points plotted.

The expression levels of *Orco* were analyzed in tissues (antennae, head, legs, and carcass) from different castes for the spatial distribution pattern, and in whole bodies of different castes or developmental stages for temporal distribution. The results showed that *Orco* was predominantly expressed in the antennae of all castes, and it was found at very low levels in other tissues (Fig 4A). *Orco* expression levels were not significantly different between the antennae of female and male alates (*P =* 0.2856, Kruskal-Wallis followed by Dunn’s test), or between worker and soldier antennae (*P =* 0.2140, Kruskal-Wallis followed by Dunn’s test). However, significantly higher expressions were found in the antennae of alates than in workers and soldiers (Fig 4A, *P <* 0.05, Kruskal-Wallis followed by Dunn’s test). A similar expression pattern was observed in the heads of all castes, where the *Orco* expression levels in alates were significantly higher than in workers (Fig 4A; worker-female alate: *P =* 0.0056; worker-male alate: *P =* 0.0480; Kruskal-Wallis followed by Dunn’s test). In the legs, the expression of *Orco* was similar in workers, female and male alates; however, the level of expression in the soldier legs was significantly lower than in female and male alates (Fig 4A, *P =* 0.0087, Kruskal-Wallis followed by Dunn’s test). For the carcass, *Orco* expression in female alates was significantly higher than that in workers (Fig 4A, *P =* 0.0056, Kruskal-Wallis followed by Dunn’s test).

The temporal expression analysis showed that *Orco* was expressed in all castes as well as the early developmental stages, such as eggs and larvae. O*rco* expression levels did not differ significantly between the whole bodies of workers and alates (female and male) (Fig 4B, *P >* 0.05, Kruskal-Wallis followed by Dunn’s test). Eggs and nymphs showed the lowest expressions of *Orco*, which were significantly lower than workers and alates of both sexes (Fig 4B; eggs-worker: *P =* 0.0126; eggs-female alate: *P =* 0.0089; egg-male alate: *P =* 0.0019; nymph-worker: *P =* 0.0106; nymph-female alate: *P =* 0.0075; nymphs-male alate: *P =* 0.0015; Kruskal-Wallis followed by Dunn’s test). The expression levels of *Orco* in larvae were not significantly different from workers, pre-soldiers, or alates of both sexes (Fig 4B, *P >* 0.05, Kruskal-Wallis followed by Dunn’s test).

### Expression of *Orco* in response to different social environments

**Fig 5:**
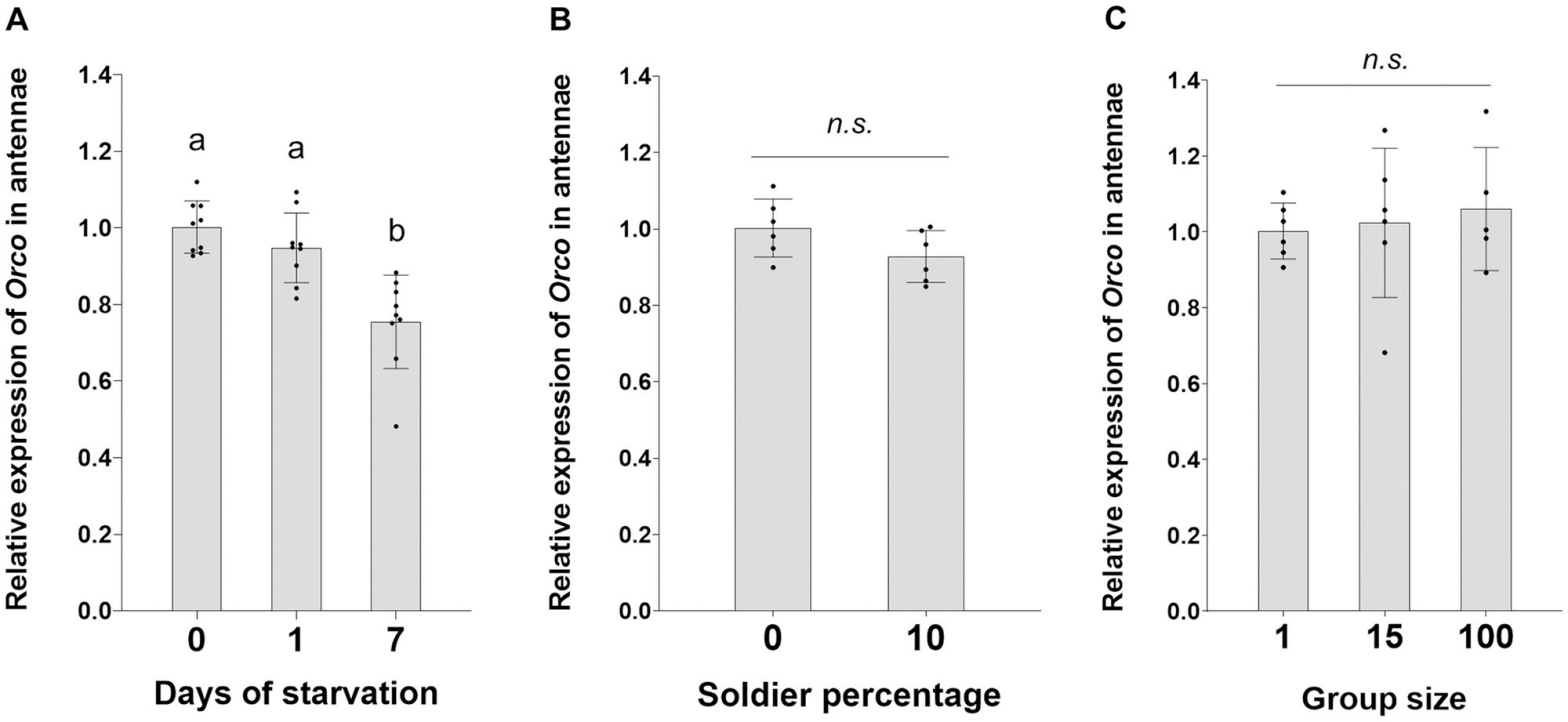
Impacts of social conditions on the expression of *Orco* in worker antennae. (A) *Orco* expression in the antennae in response to different starvation periods. (B) *Orco* expression in response to the absence and presence of soldiers in seven days. (C) *Orco* expression in response to different group sizes in seven days. Different letters denote significant differences by Kruskal-Wallis followed by Dunn’s test (*P* < 0.05, n = 9 for the starvation assay, and n = 6 for the soldier and group size assays); *n*.*s*.: not significant. In all graphs, bars represent means ± SE with individual data points plotted.

To investigate if *Orco* expression in worker antennae is affected by social conditions, termites were exposed to different levels of food deprivation, soldier percentage, and group size. In all three assays, termite mortality did not exceed 10% (S1 Fig). The results showed that the expression of *Orco* declined with increasing starvation period (Fig 5A). Workers starved for seven days had significantly lower expression of *Orco* than the ones starved for one day or not starved (Fig 5A, 0 day-1 day: *P =* 0.2380; 0 day-7 days: *P =* 0.0001; 1 day-7 days: *P =* 0.0012; Kruskal-Wallis followed by Dunn’s test).

The presence or absence of soldiers did not significantly affect the expression of *Orco* in the antennae of workers (Fig 5B; *P =* 0.1093 by Kruskal-Wallis test). Similarly, there were no significant differences in *Orco* expression among workers that were isolated as individuals or in groups of 15 or 100 (Fig 5C, *P =* 0.8809, Kruskal-Wallis test).

## Discussion

### Sequence and bioinformatics analysis of *Orco*

In this study, we identified and characterized an *Orco* ortholog in *C*. *formosanus*. This gene encodes a protein with similar length and sequence identity with orthologs from five other termite species. These species represent different termite families and ecological niches, including subterranean (Rhinotermitidae), dampwood (Termopsidae), drywood (Kalotermitidae), and fungus-growing (Termitidae) termites [28, 29, 33, 43, 44]. Our results confirm that *Orco* is highly conserved across termites. Consistent with previous studies in other insects [14], only one *Orco* is found in *C*. *formosanus*. The predicted structure of *C*. *formosanus* Orco protein is highly similar to that in *R*. *chinensis* and *O*. *formosanus* [28, 29], with seven transmembrane regions that are characteristics of the 7tm_6 protein family, and an extracellular C-terminal end. However, different from the two mentioned termite species, the prediction of *C*. *formosanus* Orco structure provides a low statistical support for the existence of an additional transmembrane region before the 7tm_6 (probability < 0.2). The 7tm_6 region and extracellular C-terminus are characteristics of odorant receptors [52, 53]. With 63.93% amino acid sequence identity, the predicted 3D structure of *C*. *formosanus* Orco exhibited similar membrane topology with that in the fig wasp *A*. *bakeri* characterized by cryo-electron microscopy [15].

Our phylogenetic reconstruction supports the high conservation of *Orco* across insect taxa, and Orco proteins of Blattodea are evolutionary closer to Orthoptera than other orders. While the divergent *OR* family confers odorant specificity and co-varies with the chemical ecology of insects, *Orco* is considered as one of the most conserved genes in insects in terms of sequence, protein structure, and function [52, 54].

### Spatial and temporal expression of Orco

The gene expression analysis revealed that *C*. *formosanus Orco* is primarily expressed in the antennae (Fig 4A), the main olfactory organ for the expression of most *OR*s in insects [12]. It is worth noting that the antennal expression of *Orco* is significantly higher in alates than workers and soldiers (Fig 4A). This result is consistent with our previous morphological analysis that alates of both sexes possess longer antennae and higher numbers of antennal sensilla than the non-reproductive castes [32]. The discrepancy implies differential olfactory sensitivity between reproductive and non-reproductive individuals in *C*. *formosanus*. Compared with other individuals, alates have an expanded behavioral repertoire, which may rely on perception of a wider range of olfactory cues. Dispersal alates are exposed to the open environment, and they must detect the sex pheromone from mates and a suitable nest site for successful colony foundation [55, 56]. During the incipient stage of a colony, chemical signals are expected to mediate brood care and nestmate recognition by the young queens and kings [57, 58]. Workers and soldiers, by contrast, live in enclosed underground nests and perform collective behavior related to foraging, colony hygiene, and defense [5, 6]. The increase in *Orco* expression in alates is likely associated with the need for dispersal and mating. Antennal expression of *Orco* did not differ between workers and soldiers, despite the behavioral differences in the two castes. Additional olfactory mechanisms, such as ORs and neural circuits, warrant further investigation to understand the division of labor in termites.

In *C*. *formosanus*, *Orco* is expressed in all developmental stages, including eggs, larvae, pre-soldiers, and nymphs at varying levels (Fig 4B). Low expression of *Orco* in eggs and larvae has also been reported in holometabolous insects, such as the oriental fruit fly (*Bactrocera dorsalis*) [41] and the common green bottle fly (*Lucilia sericata*) [59]. Due to the tissue-specific distribution of *Orco* and body size variation among the developing individuals, the expression level of *Orco* in whole-body samples does not necessarily reflect olfactory sensitivity. The expression of *Orco* during early development, such as eggs and larvae, may be important for proper development of olfactory processing neurons in the brain. The expression of *Orco* is required for the development of antennal lobe glomeruli in several species of social Hymenoptera, including the honey bee (*A*. *mellifera*) [26] and two ants (*O*. *biroi* and *H*. *saltator*) [24, 25]. In *Drosophila*, Orco is fundamental for proper trafficking and structural localization of ORs in the outer ciliated dendrites of OSNs [18], which is fundamental for proper activity and responsiveness of these neurons to odorants. In *C*. *formosanus* eggs and larvae, *Orco* expression is possibly needed for proper structural arrangement of ORs in the developing OSNs. However, this hypothesis is yet to be tested.

In this study, RNA samples from the head, carcass, and whole body of soldiers were not obtained due to technical issues caused by defensive chemicals stored in their frontal gland. In *C*. *formosanus* soldiers, the frontal gland extends from head to abdomen, containing abundant lignoceric acid, hexacosanoic acid, and other lipids in aqueous mucopolysacharides [60, 61]. Additionally, the sticky fluid is enclosed in very thin epithelium (8–16 μm) [62], which makes removal of the intact gland from soldiers technically challenging.

### Expression of *Orco* in response to different social environments

In addition to environmental conditions during postembryonic development, olfactory plasticity in insects can also be modulated by the immediate sensory environment. The plastic changes allow insects to respond to the chemical surroundings according to their physiological and behavioral state [35]. In *C*. *formosanus* workers, starvation for seven days downregulated *Orco* expression in the antennae, while this effect was not detected for one-day starvation (Fig 5A). Food availability is an important environmental factor that mediates foraging behavior in subterranean termites. Compared to conditions when different food sizes were provided, food deprivation was observed to promote exploratory tunneling behavior in *C*. *formosanus* [63], whereas the role olfaction plays in the process remains unclear. Generally, starvation enhances olfactory sensitivity in insects through changes in chemosensory gene expression for odorant perception and neuropeptides for olfactory processing [35, 39, 40, 64, 65]. Consistent with our observations of *C*. *formosanus*, *Drosophila* larvae exhibited a decrease in *Orco* expression due to decreased activity in the insulin signaling pathway after starvation, although starvation enhanced their olfactory behavior toward certain odorants [65]. Nutritional effects on *Orco* and olfactory sensitivity have also been demonstrated in adult *D*. *melanogaster* [66]. Feeding on a high fat diet for 14 days decreased olfactory sensitivity in the flies, which was correlated with a decreased expression of *Orco*, along with reduced expression of the insulin-like peptide 2 and the insulin receptor, as well as nine upregulated and 21 downregulated odorant binding proteins (OBPs) [66]. These studies suggest that *Orco* is involved in nutrition-dependent olfactory behavior by downstream modulation of insulin signaling in OSNs and through complex interactions with other chemosensory genes. The reduced *Orco* expression in *C*. *formosanus* workers does not necessarily indicate decreased olfactory sensitivity after starvation, as ORs, OBPs, and other chemosensory proteins are likely involved in olfactory regulation and may affect the overall sensitivity. The consequences of starvation on olfaction and the regulatory mechanisms await further investigation in termites.

Our results showed that *Orco* expression in workers was not affected by the absence of soldiers, suggesting soldier cue does not influence short-term (seven day) olfactory capacity of *C*. *formosanus* workers (Fig 5B). In termites, workers and soldiers both constitute the foraging population, and 10% soldiers are generally found in *C*. *formosanus* colonies [67]. In *R*. *flavipes*, the presence of soldiers provided a social buffering effect, which altered worker behavior and reduced their mortality induced by predator-induced stress in two days [68]. In this study, the absence of soldiers without environmental stress did not influence worker *Orco* expression in seven days, but it needs to be determined whether there is a longer-term effect. In the absence of soldiers, *C*. *formosanus* workers differentiate into soldiers through an intermediate pre-soldier stage. Previous studies showed that this transition took 35 days or longer, and the differentiation was triggered by increased juvenile hormone (JH) titers in workers and dependent on group size [69]. Many genes in the JH signaling and insulin pathways are involved in the caste transition [70], but changes in olfaction-related gene expression during this process and their roles in worker-soldier interactions require further investigation.

In social insects, colony functions are achieved through collective activities and interactions among colony members, and isolation from the social group affects individual fitness [24]. In *C*. *formosanus*, group size is a social factor regulating the self-organized tunneling activity during foraging, and tunnel construction is positively correlated with the density and flow rate of individuals through nestmate interactions, presumably based on olfaction and/or mechanical stimuli [71, 72]. Our study in *C*. *formosanus* showed that isolated workers did not differ in their antennal expression of *Orco* compared with those in groups of 15 or 100 (Fig 5C). Similarly, in the silky ant, *Formica fusca*, *Orco* was not differentially expressed between isolated larvae and those exposed to social cues, while many other chemosensory genes, such as *OR*s and *OBP*s, were upregulated upon social stimulation [73]. Taken together, while interactions with soldiers and nestmate workers are both important for the social life of termites, social cues indicating soldier presence and group size did not alter *Orco* expression in *C*. *formosanus* workers. As *Orco* is crucial for all aspects of olfactory activity, its stable expression might be important for maintaining the basic olfactory function in termites, which enables rapid behavioral response to the dynamic social environment through the changes of other odorant-specific chemosensory genes. Olfactory plasticity via changes in *Orco* expression is likely associated with physiological changes as induced by starvation and during caste differentiation. Future studies of additional mechanisms, such as ORs and neurotransmitters, are necessary to better understand the short-term fine tuning of olfaction-related behavior in termites.

## Supporting information

S1 Table Primers used in this study. (DOCX)

S2 Table Accession numbers of the *Orco* gene from different insect species utilized for phylogenetic analysis. (DOCX)

S3 Table Descriptive statistical results for the reference genes evaluated with BestKeeper. (DOCX)

S1 Fig Termite survivorship in gene expression analyses. (DOCX)

S1 Data Original data used in this study. (XLSX)
