# Supplementary material for: Molecular characterization and expression variation of the odorant receptor co-receptor in the Formosan subterranean termite
Source: PLoS One. 2022 Apr 28;17(4):e0267841. doi: 10.1371/journal.pone.0267841 (PMC9049313; doi:10.1371/journal.pone.0267841)
Supplement: S1 Table — (DOCX) [file pone.0267841.s001.docx]

**S1 Table.** Primers used in this study.

| **Gene Name** | **Orientation** | **(5′→3′) Primer Sequence** | **Purpose** |
| --- | --- | --- | --- |
| *Orco* | 3GSP | TTCCTGGCCCTGTCGCACGCCATGATGA | full-length amplification |
| *Orco* | 5GSP | TTGCTCCTCCGACCCGTCATACCACTGACAGC | full-length amplification |
| *Orco* | Forward | TGCCTGCACAGTAATCGGCTA | qRT-PCR |
|  | Reverse | CAGCCTCCATCACTGACGA |  |
| *rps18* | Forward | CAGCCTCCATCACTGACGA | qRT-PCR |
|  | Reverse | CCCGCTTATCCAGATCAATGT |  |
| *rpl32* | Forward | ATGGAGGAAACCTAAGGGTATTG | qRT-PCR |
|  | Reverse | AAGCCAGTAGGAAGCATGTG |  |
| *ef1-α* | Forward | ATTGAGCGTAAGGAGGGTAAAG | qRT-PCR |
|  | Reverse | CATCCTGAAGAGGAAGACGAAG |  |
| *β-Actin* | Forward | GACATCAAAGAGAAACTGTGCTATG | qRT-PCR |
|  | Reverse | ACCATCAGGCAACTCGTATG |  |
