# Supplementary material for: Molecular characterization and expression variation of the odorant receptor co-receptor in the Formosan subterranean termite
Source: PLoS One. 2022 Apr 28;17(4):e0267841. doi: 10.1371/journal.pone.0267841 (PMC9049313; doi:10.1371/journal.pone.0267841)
Supplement: S2 Table — (DOCX) [file pone.0267841.s002.docx]

**S2 Table.** Accession numbers of the *Orco* gene from different insect species utilized for phylogenetic analysis.

| **Insect order** | **Common name** | **Scientific name** | **NCBI Accession number** |
| --- | --- | --- | --- |
| Blattodea | American cockroach | *Periplaneta Americana* | BDC30331.1 |
| Blattodea | German cockroach | *Blattella germanica* | PSN39983.1 |
| Blattodea | Formosan subterranean termite | *Coptotermes formosanus* | OL845867 |
| Blattodea | Drywood termite | *Cryptotermes secundus* | XP_023716643.1 |
| Blattodea | Fungus-growing termite | *Odontotermes formosanus* | QZA87370.1 |
| Blattodea | Chinese subterranean termite | *Reticulitermes chinensis* | MK940559.1 |
| Blattodea | Japanese subterranean termite | *Reticulitermes speratus* | BAU20240.1 |
| Blattodea | Dampwood termite | *Zootermopsis nevadensis* | XP_021933609.1 |
| Coleoptera | Yellow mealworm | *Tenebrio molitor* | AJO62219.1 |
| Coleoptera | Red flour beetle | *Tribolium castaneum* | XP_008194693.1 |
| Coleoptera | Black flour beetle | *Tribolium madens* | XP_044261477.1 |
| Diptera | Yellow fever mosquito | *Aedes aegypti* | NP_001345400.1 |
| Diptera | Asian tiger mosquito | *Aedes albopictus* | AHL20247.1 |
| Diptera | Fruit fly | *Drosophila melanogaster* | NP_524235.2 |
| Diptera | Spotted wing Drosophila | *Drosophila suzukii* | XP_036675168.1 |
| Diptera | House fly | *Musca domestica* | AFH96944.1 |
| Hemiptera | Pea aphid | *Acyrthosiphon pisum* | AQS60741.1 |
| Hemiptera | Asia citrus psyllid | *Diaphorina citri* | QPZ88913.1 |
| Hemiptera | Bed bug | *Cimex lectularius* | NP_001303637.1 |
| Hymenoptera | Honey bee | *Apis mellifera* | XP_006563267.1 |
| Hymenoptera | Fig wasp | *Apocryta bakeri* | 6C70_A |
| Hymenoptera | Jerdon’s jumping ant | *Harpegnathos saltator* | XP_011139767.1 |
| Hymenoptera | Clonal raider ant | *Ooceraea biroi* | XP_011346854.1 |
| Lepidoptera | Cotton bollworm | *Helicoverpa armigera* | ADQ13177.1 |
| Lepidoptera | Corn Earworm | *Helicoverpa zea* | AAX14773.1 |
| Lepidoptera | Fall Armyworm | *Spodoptera frugiperda* | XP_035448751.1 |
| Orthoptera | Migratory locust | *Locusta migratoria* | AEX28370.1 |
| Orthoptera | Desert locust | *Schistocerca gregaria* | AEX28371.1 |
| Orthoptera | Grasshopper | *Oedaleus asiaticus* | QAB43939.1 |
