# Supplementary material for: Molecular characterization and expression variation of the odorant receptor co-receptor in the Formosan subterranean termite
Source: PLoS One. 2022 Apr 28;17(4):e0267841. doi: 10.1371/journal.pone.0267841 (PMC9049313; doi:10.1371/journal.pone.0267841)
Supplement: S3 Table — (DOCX) [file pone.0267841.s003.docx]

**S3 Table**. Descriptive statistical results for the reference genes evaluated with BestKeeper. The stability of four reference genes were tested across all samples. *rps18* and *rpl32* were both stable in all samples with comparable coefficients of variance. *ef1-α* and *β-Actin* were less stable and exceeded the 3-fold overexpression threshold in the analyses of tissues and castes. In the analyses of castes and group size, all reference genes had a standard deviation higher than 1.0, but *rps18* and *rpl32* were the genes with less variation in the caste samples. To determine the relative gene expression levels of *Orco* in this study, *rps18* was selected as the reference gene.

| Gene expression analysis | Reference  gene | n | Geo Mean  [Cq] | Ar Mean  [Cq] | Min  [Cq] | Max  [Cq] | Std dev  [± Cq] | CV  [% Cq] | Min  [x-fold] | Max  [x-fold] | Std dev  [± x-fold] |
| --- | --- | --- | --- | --- | --- | --- | --- | --- | --- | --- | --- |
| Tissues | *rps18* | 68 | 21.93358 | 21.97048 | 20.31385 | 25.08531 | 1.097051 | 4.993296 | -2.91059 | 7.995317 | 2.061852 |
|  | *rpl32* | 68 | 21.65119 | 21.67629 | 20.04459 | 24.54613 | 0.808189 | 3.728448 | -2.88549 | 6.749551 | 1.704163 |
|  | *ef1-α* | 68 | 19.22438 | 19.25535 | 17.74401 | 22.23700 | 0.869658 | 4.516447 | -2.65499 | 7.294315 | 1.774676 |
|  | *β-actin* | 68 | 18.31484 | 18.48139 | 14.67794 | 22.60847 | 2.336931 | 12.64478 | -11.0106 | 16.98001 | 4.671204 |
| Castes | *rps18* | 44 | 22.32476 | 22.37806 | 20.45230 | 25.31845 | 1.348419 | 6.025632 | -3.72454 | 8.185252 | 2.577797 |
|  | *rpl32* | 44 | 22.12894 | 22.17911 | 20.45969 | 25.13382 | 1.319266 | 5.948237 | -3.22923 | 8.249792 | 2.525557 |
|  | *ef1-α* | 44 | 20.38080 | 20.48929 | 17.74413 | 24.10406 | 1.934305 | 9.440567 | -6.37009 | 13.66289 | 3.889872 |
|  | *β-actin* | 44 | 19.73464 | 19.88715 | 16.60786 | 23.65345 | 2.265707 | 11.39282 | -8.98720 | 15.67400 | 4.909174 |
| Starvation | *rps18* | 54 | 22.70765 | 22.7341 | 20.72773 | 24.92788 | 0.867793 | 3.817138 | -3.65346 | 4.275641 | 1.764538 |
|  | *rpl32* | 54 | 22.78216 | 22.8133 | 20.68332 | 25.14568 | 0.929099 | 4.072603 | -3.93192 | 4.672909 | 1.836769 |
|  | *ef1-α* | 54 | 20.50846 | 20.5434 | 18.30661 | 22.81702 | 0.942429 | 4.587493 | -4.22452 | 4.530073 | 1.852862 |
|  | *β-actin* | 54 | 25.60528 | 25.6342 | 23.56497 | 28.33369 | 0.956129 | 3.729895 | -3.80071 | 5.962489 | 1.869548 |
| Soldier presence | *rps18* | 24 | 22.77651 | 22.7887 | 21.1932 | 23.88642 | 0.626641 | 2.749787 | -2.76513 | 2.040067 | 1.495621 |
|  | *rpl32* | 24 | 23.15127 | 23.1620 | 21.7688 | 24.32569 | 0.594309 | 2.565881 | -2.43034 | 2.126381 | 1.464879 |
|  | *ef1-α* | 24 | 20.88239 | 20.8957 | 19.5716 | 22.07159 | 0.660861 | 3.162655 | -2.32106 | 2.146670 | 1.528862 |
|  | *β-actin* | 24 | 25.56901 | 25.5841 | 23.7069 | 27.46129 | 0.667421 | 2.608726 | -3.30726 | 3.372185 | 1.535319 |
| Group size | *rps18* | 36 | 23.7363 | 23.81901 | 20.3222 | 28.01828 | 1.644130 | 6.902596 | -8.51274 | 14.6725 | 2.804768 |
|  | *rpl32* | 36 | 24.4569 | 24.53304 | 21.6211 | 28.93331 | 1.600598 | 6.524254 | -5.92294 | 16.5756 | 2.729215 |
|  | *ef1-α* | 36 | 21.2918 | 21.36912 | 18.7193 | 25.63716 | 1.539645 | 7.204998 | -5.02107 | 15.2672 | 2.626835 |
|  | *β-actin* | 36 | 26.1810 | 26.24296 | 23.2534 | 30.42872 | 1.507433 | 5.744144 | -6.27408 | 14.3602 | 2.574291 |

n: number of samples; Geo Mean [Cq]: geometric mean of Cq; Ar Mean [Cq]: arithmetric mean of Cq; Min [Cq] and Max [Cq]: minimum and maximum values of Cq; Std dev [± Cq]: standard deviation of the Cq; CV [%Cq]: coefficient of variance expressed as a percentage on the Cq level; Min [x-fold] and Max [x-fold]: minimum and maximum values of expression levels expressed as an absolute x-fold over- or under-regulation coefficient; Std dev [± x-fold]: standard deviation of the absolute regulation coefficients.
