# Supplementary material for: Molecular characterization and expression variation of the odorant receptor co-receptor in the Formosan subterranean termite
Source: PLoS One. 2022 Apr 28;17(4):e0267841. doi: 10.1371/journal.pone.0267841 (PMC9049313; doi:10.1371/journal.pone.0267841)
Supplement: S1 Fig — (DOCX) [file pone.0267841.s004.docx]

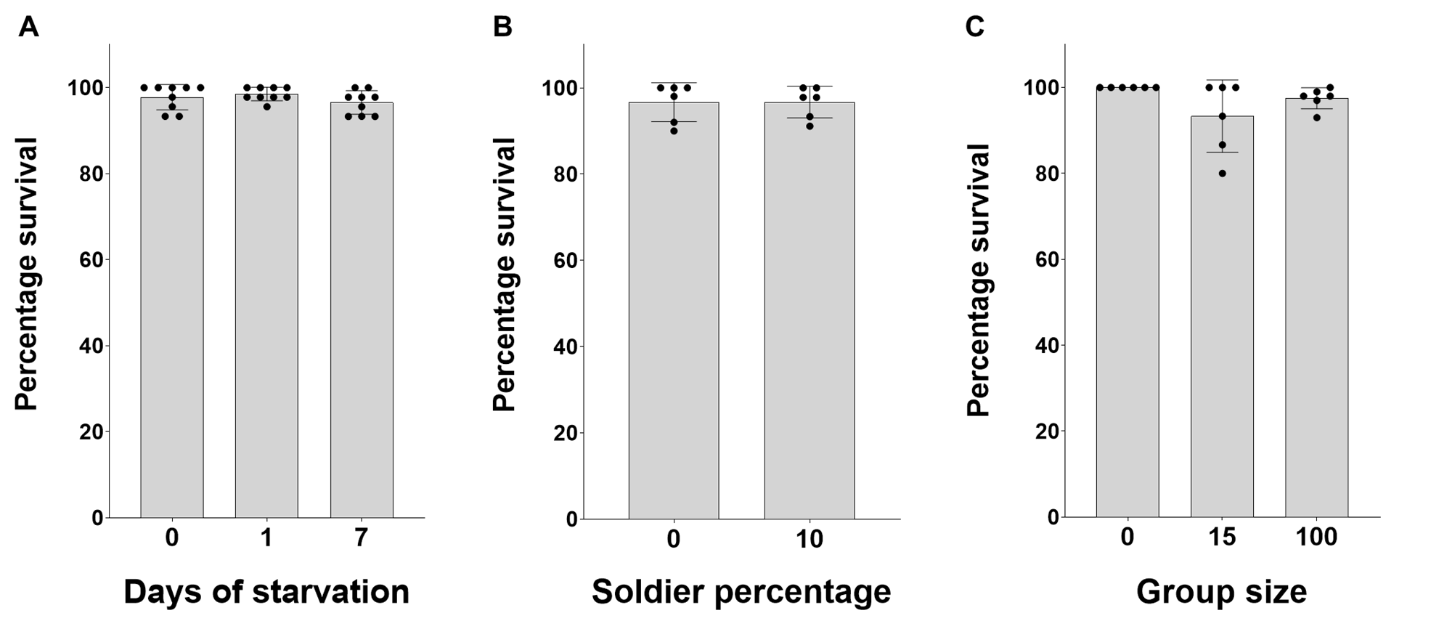


**S1 Fig.** Termite survivorship in gene expression analyses after seven days of treatment in response to (A) starvation, (B) soldier percentage, and (C) group size. Bars show mean ± SE with individual data points plotted; n = 9 per group in the starvation analysis; and n = 6 per group in the soldier percentage and group size analyses.
